# Foot orthoses for first metatarsophalangeal joint osteoarthritis: study protocol for the FORT randomised controlled trial

**DOI:** 10.1186/s12891-020-03809-x

**Published:** 2020-12-10

**Authors:** Kade L. Paterson, Rana S. Hinman, Ben R. Metcalf, Sarah E. Jones, Hylton B. Menz, Shannon E. Munteanu, Jessica Kasza, Kim L. Bennell

**Affiliations:** 1 Centre for Health, Exercise and Sports Medicine, Department of Physiotherapy, School of Health Sciences, Faculty of Medicine Dentistry & Health Sciences, The University of Melbourne, Melbourne, Australia; 2 School of Allied Health, Human Services and Sport, La Trobe University, Melbourne, Victoria Australia; 3 School of Public Health and Preventive Medicine, Monash University, Melbourne, Australia

**Keywords:** Osteoarthritis, OA, Foot, Metatarsophalangeal, Orthoses, Insoles, Clinical trial, RCT, Biomechanics, Pain

## Abstract

**Background:**

First metatarsophalangeal (MTP) joint osteoarthritis (OA) is a painful and debilitating condition affecting nearly one in 10 people aged over 50 years. Non-drug, non-surgical treatments are recommended by OA clinical guidelines, yet there have only ever been two randomised controlled trials (RCTs) evaluating such strategies in people with first MTP joint OA. Foot orthoses are a common non-drug, non-surgical strategy used by allied health professionals for people with first MTP joint OA, however, it is unknown whether these devices are effective in improving the symptoms associated with the condition. This clinical trial aimed to determine whether contoured foot orthoses lead to greater reductions in first MTP joint pain on walking compared to sham flat insoles in people with first MTP joint OA.

**Methods:**

The FORT trial (Foot ORthoses for big Toe joint osteoarthritis) is a two-arm participant- and assessor-blinded, multi-site RCT conducted in Melbourne, Sydney, Brisbane and the Gold Coast, Australia. We are recruiting 88 community-dwelling people with symptomatic radiographic first MTP joint OA. Following baseline assessment, participants are randomized to receive either: i) contoured foot orthoses; or ii) sham flat insoles following baseline assessment. Participants have two visits with a study podiatrist where they are provided with their allocated insoles, to be worn daily for 12 weeks at all times when wearing shoes. The primary outcome is self-reported first MTP joint pain on walking (numerical rating scale), assessed at baseline and 12 weeks. Secondary outcomes include additional measures of first MTP joint and foot pain, physical function, quality of life, participant-perceived global ratings of change (pain and function), and level of physical activity.

**Discussion:**

This study will provide novel evidence about whether contoured foot orthoses improve pain and other symptoms compared to sham insoles in people with first MTP joint OA. Outcomes will help to inform clinical guidelines and practice about the use of foot orthoses for managing symptoms in this under-researched group of people with OA.

**Trial registration:**

Prospectively registered with the Australian New Zealand Clinical Trials Registry (reference: ACTRN12619000926134) on 3/07/2019.

## Background

Osteoarthritis (OA) of the foot affects 16.7% of people aged 50 years and older, making it as prevalent as knee OA [1], and the most commonly affected foot site is the first metatarsophalangeal (MTP) joint [2]. First MTP joint OA is highly debilitating with most (72%) of those affected reporting the symptoms as disabling [2]. The condition causes dorsal osteophytes and joint space narrowing which impair range of motion, and in turn, lead to substantial problems performing functional weight bearing activities and impaired quality of life [3]. Non-drug, non-surgical strategies are recommended as first line treatments in international OA clinical guidelines [4–7], however there have only ever been two randomised controlled trials (RCTs) of these treatments for first MTP joint OA [8, 9], and one randomized clinical feasibility study [10]. Accordingly, foot OA has been highlighted as an under-researched problem [11] and identified as a research priority by OA experts and all leading international OA clinical bodies [4, 12, 13].

People with first MTP joint OA walk with altered foot biomechanics, displaying reduced joint range of motion and greater maximum force and peak plantar pressures under the hallux [14–16], which may contribute to symptom severity. Foot orthoses with a cut-out under the first MTP joint have been shown to reduce hallux plantar pressures in people with first MTP joint OA [17] and improve the first metatarsal angle and MTP joint range of motion in people with limited first MTP joint movement [18]. Furthermore, a case series has shown they may have beneficial effects on pain in a small group of people with mechanically induced first MTP joint pain [19], suggesting these biomechanical devices may improve clinical symptoms associated with first MTP joint OA. We have also shown that foot orthoses are commonly used in clinical practice. Results of our international survey of podiatrists and physiotherapists who currently treat people with first MTP joint OA showed that foot orthoses were one of the most commonly employed treatment strategies to manage the condition [20].

To date, only one RCT has investigated whether foot orthoses are effective in treating the symptoms associated with first MTP joint OA. Menz and colleagues reported that foot orthoses and rocker-sole footwear both improved pain above the minimal clinically important difference, with no statistically significant difference between the two interventions [9]. However, the use of an active comparator as the control condition in this study means it is unclear whether the clinical improvements seen in both treatment groups were due to true effects of the interventions, placebo effects, or to other factors such as natural history or regression to the mean. The need for such research has also been highlighted by OA stakeholders and patients, who have rated non-surgical treatment and biomechanical strategies within their top 10 OA research priorities [21].

This study protocol describes a clinical trial that compares the effects of contoured foot orthoses and sham flat insoles in people with symptomatic radiographic first MTP joint OA. The primary aim is to determine whether daily wear of contoured foot orthoses results in significantly greater reductions in first MTP joint pain with walking over 12 weeks compared to sham flat insoles, in people with first MTP joint OA. The secondary aim is to determine if contoured foot orthoses improve additional measures of pain, function, quality of life and physical activity when compared to sham flat insoles, at 12 weeks.

## Methods

### Study design

The FORT (Foot ORthoses for big Toe joint osteoarthritis) RCT is a participant- and assessor-blinded trial, conducted at sites in Melbourne, Sydney, Brisbane and the Gold Coast in Australia. It was prospectively registered with the Australian and New Zealand Clinical Trials Registry (ACTRN12619000926134) and ethical approval has been obtained from the University of Melbourne Human Research Ethics Committee (HREC No. 1954551). This protocol paper is described using the 2013 SPIRIT guidelines on standard protocol items for clinical trials [22].

### Participants

Community-dwelling participants with symptomatic radiographic first MTP joint OA are recruited via advertisements in print and social media, and using our clinician network and volunteer database. As there are no accepted clinical diagnostic criteria for first MTP joint OA, we are using National Institute for Health and Care Excellence OA criteria [4] combined with radiographic features of first MTP joint OA [23]. Participants are eligible if the following inclusion criteria are met:

i) aged > 45 years,
ii) report first MTP joint pain on most days of the last month and for at least 12 weeks,
iii) report a minimum pain score of 3 on an 11-point numerical rating scale during walking over the previous week,
iv) evidence of radiographic OA (a score of 2 or more for osteophytes or joint space narrowing according to a radiographic atlas [23]), and
v) report either no morning joint-related stiffness in the first MTP joint, or morning stiffness that lasts no longer than 30 min.

Participants are excluded if they:

i) have had previous foot surgery on the affected side or are planning surgery in the next 12 weeks,
ii) currently use foot orthoses or ankle braces,
iii) currently primarily use high heels, sandals or other shoes that would restrict ability to wear orthoses,
iv) have had any foot injections on the affected side in the past 6 months or are planning injections in the next 12 weeks,
v) report any other current muscular, joint or neurological condition affecting lower limb function,
vi) have pain in another location that is greater than the pain in the study first MTP joint,
vii) report any systemic or inflammatory joint disease (eg rheumatoid arthritis),
viii) have grade 3 or 4 hallux valgus on the affected side [24],
ix) currently use or are planning to use a gait aid in the next 12 weeks,
x) are unable to understand written/spoken English,
xi) are unable to commit to study requirements (eg wearing insoles, attending appointments, completing outcomes).

### Procedure

**Fig. 1:**
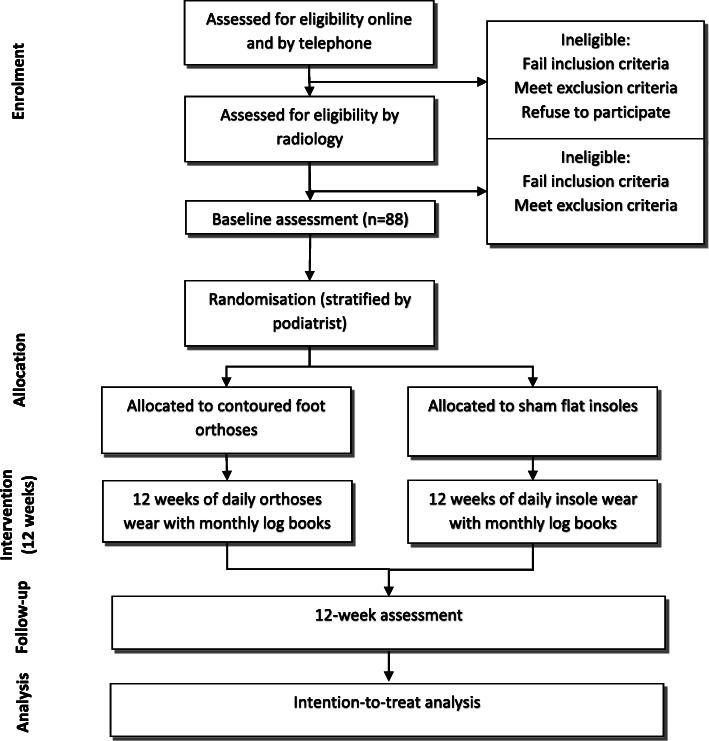
Flow diagram of s tudy phases

Figure 1 outlines the flow of participants through the trial and Table 1 describes the schedule of enrolment, interventions and the outcome measures for this study, according to SPIRIT recommendations [22]. Potential volunteers receive study information that describes the aims, potential risks and protocols involved. Volunteers are screened by an online form, then over the phone by the Trial Coordinator. All participants then provide informed consent. Potentially eligible participants undergo standardised dorsoplantar and lateral weightbearing x-rays at radiology clinics in Melbourne, Sydney, Brisbane or the Gold Coast. Participants who have had a previous (past 12 months) weightbearing dorsoplantar and lateral x-ray, and can provide the images, do not have new x-rays due to ethical concerns of exposure to unnecessary radiation. X-rays are assessed for eligibility by an experienced researcher (KP). For participants with bilaterally eligible feet, the most symptomatic foot is used as the study foot for outcome measurements.

**Table 1:**
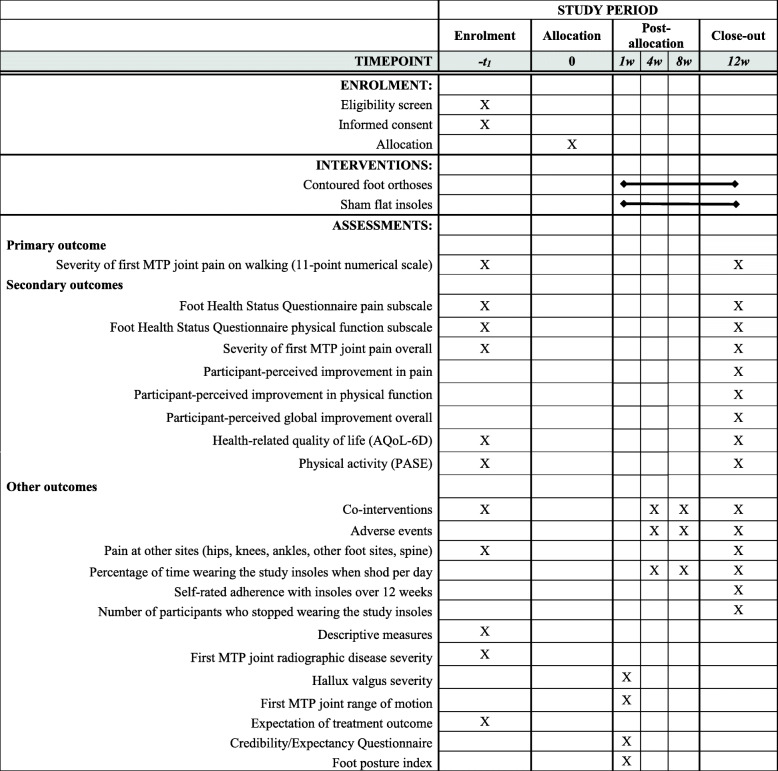
Schedule of enrolment, interventions and assessments

*W* week, *MTP* metatarsophalangeal, *AQoL-6D* Assessment of Quality of Life instrument (version 6D), *PASE* physical activity scale for the elderly

Paper-based or electronic baseline assessments are completed by participants at home. Once the baseline questionnaire has been completed and returned to the researchers, participants are enrolled in the study and randomly allocated to one of the two study groups. After enrolment, a researcher contacts the participant to arrange their two appointments with their podiatrist where they are provided with a pair of their allocated insoles. The participant is advised to choose a participating podiatry clinic that is most convenient for them. Self-reported outcomes are completed at the participant’s home either electronically or on paper at 12-weeks.

### Randomisation, blinding and allocation concealment

Participants are randomised to either the contoured foot orthoses group or the sham flat insoles group using a randomisation schedule prepared by the study biostatistician. Randomisation occurs on a 1:1 allocation with permuted block sizes of 6 to 12, stratified by podiatrist/clinic. The schedule is uploaded and stored on a password-protected website (REDCap) where access is limited to a researcher not involved with participant recruitment or outcome measure administration to ensure concealment. Group allocation is revealed by a different researcher not involved in either participant recruitment or data collection after baseline outcomes have been completed.

Limited disclosure is used to blind participants to group allocation. Participants are told that this clinical trial is comparing two types of insoles but they are not informed about the characteristics of the insoles provided to either group, consistent with our previous insole and footwear OA trials [25, 26]. Participants are also not informed about the specific aims or hypotheses of the study until after the study is completed. At this time, participants will be given a summary outlining the study purpose and main findings. Primary and secondary outcomes are participant-reported, thus this study is also assessor-blinded (because participants are blinded). Study researchers entering participant-reported data will be blinded, as will the study biostatistician performing the statistical analyses. The podiatrists administering the insoles will not be blinded, which is a recognised difficulty when conducting trials using a physical intervention [27]. Stratification by podiatrists/clinics will ensure each podiatrist will treat a similar proportion of participants in both groups to ensure that the effects of podiatrist personality on the therapeutic alliance are consistent across groups.

### Insole interventions

Table 2 describes the contoured foot orthoses and the sham flat insoles. Participants in both groups have two visits with a study podiatrist, one in week 1 and one in week 3. The initial consultation is 30 min and the follow-up visit is 15-min.

**Table 2:** Intervention and control insoles

Contoured foot orthoses (intervention)	Sham flat insole (control)
Blue Formthotic^TM^ (Foot Science International) Blue, medium density (140 kg/m^3^ single-density, closed-cell polyethylene foam) prefabricated Formthotic^TM^ which possesses heel and arch support. A first MTP joint cut out is added if tolerated, and a blue 4°medium density varus wedge is added if the Foot Posture Index score is >7.	Blue flat insole (Foot Science International) Blue, medium density (140 kg/m^3^ single-density, closed-cell polyethylene foam) 3mm flat insole.

Images taken from Bonanno et al. [28]

The intervention group receives foot orthoses, protocolised as outlined below to ensure consistency (whilst allowing some individual variation) based on findings from our international survey [20], and developed by expert consensus during our prior feasibility RCT [10]. Specifically, a blue (medium-density) Formthotic™ prefabricated foot orthotic (Foot Science International, Christchurch, New Zealand) is prescribed, and modified using a first MTP joint cut out for joint rotation. If the participant does not have any available first MTP joint range of motion (i.e. joint fusion), or if the cut out elicits immediate pain on walking, then a first ray extension is used instead. Podiatrists score the foot posture index (FPI; a reliable and valid measure of foot posture – see below [29]) and participants with an FPI score > 7 also have a full length 4° varus wedge (Formthotics, Foot Science International, Christchurch, New Zealand) adhered to the plantar surface of the orthoses. Participants in the control group are provided with a sham flat 3 mm prefabricated insole that is the same colour and density and with the same branding as the contoured foot orthoses, but without any heel or arch support. Orthoses/insoles are trimmed (if needed) and fitted into participant’s footwear at the initial visit. If appropriate, the podiatrist will provide advice regarding use of footwear with a wide, deep toe box that is able to accommodate orthoses. If the participant did not bring appropriate footwear that can accommodate the orthoses, they will be encouraged to bring an appropriate pair to the next consultation. At this time, participants are advised to initially commence wearing the orthoses/insoles for 1 h on the first day and to increase use by an additional hour each day, until they are wearing them at all times when shod for 12 weeks. The orthoses/insoles are reviewed at the follow up visit, and additional modifications to address comfort or adverse events (such as trimming the length) may be provided (and documented), if necessary.

Eight podiatrists (three in Melbourne, three in Sydney, one in Brisbane and one in the Gold Coast) were recruited from our clinical networks. The study podiatrists have a minimum of 2 years’ experience treating people with first MTP joint OA. Podiatrists were sent a study manual and completed a one-hour online training session where they were trained in study protocols. Podiatrists were also sent treatment notes which outline each step of the intervention, and which they are required to complete for each participant, to ensure trial fidelity.

## Outcome measures

### Primary outcome

#### First MTP joint pain on walking

Overall average pain intensity on walking in the last week is assessed at baseline and 12-weeks using an 11-point numerical rating scale (NRS) with terminal descriptors of ‘no pain’ (score = 0) and ‘worst pain possible’ (score = 10). This was chosen because it has well established clinometric properties in OA [30] and is a recommended outcome measure for OA RCTs [31].

### Secondary outcomes

#### Foot pain

Foot pain over the past week is measured using the pain subscale of the Foot Health Status Questionnaire (FHSQ), a foot-specific tool with high internal consistency and test-retest reliability [32]. This subscale contains 4 questions related to overall foot pain, each rated on a 5-point Likert scale from 0 (none) to 4 (severe). Responses are recoded to provide a score between 0 (worst foot health) to 100 (optimal foot health).

#### Physical function

The function subscale of the FHSQ is used to assess physical function over the previous week [32]. This subscale contains 4 questions related to how much foot pain interferes with specific activities, each rated on a 5-point Likert scale from 0 (not at all) to 4 (extremely). Responses are recoded to provide a score between 0 (worst foot function) to 100 (best foot function).

#### Overall first MTP joint pain

Overall average pain intensity in the last week is scored on an 11-point NRS, with terminal descriptors ‘no pain’ (score = 0) and ‘worst pain possible’ (score = 10).

#### Participant-perceived change

Participants rate their change in i) pain; ii) function; and iii) overall (from baseline) over the 12 weeks of the study via a 7-point Likert scale (from ‘much worse’ to ‘much better’) [33]. Participants are classified as improved if they report being ‘moderately better’ or ‘much better’.

#### Health-related quality of life

Health-related quality of life is measured using the Assessment of Quality of Life (AQoL-6D) instrument, which has strong psychometric properties and is more responsive than other widely-used scales [34]. The AQoL-6D contains 20 items assessing independent living, mental health, relationships, pain, coping and senses. Scores range from − 0.04 to 1.00, with higher scores indicating better quality of life.

#### Physical activity

Physical activity over the previous week is measured using the Physical Activity Scale for the Elderly (PASE) [35] which has been validated against accelerometry [36]. Scores range from 0 to > 400, where higher scores indicate more physical activity.

### Other measures

#### Co-interventions

Participants self-report visits to health care providers, use of prescription and over the counter medication, hospitalisation and/or investigative procedures over the previous 7 days in monthly log books, and over the previous 12 weeks via a custom survey at baseline and 12 weeks.

#### Adverse events

Adverse events, defined as any problem experienced in the study first MTP joint or other body region due to wearing the study insole, are recorded by podiatrists in treatment notes, self-reported in log-books and ascertained by open-ended questioning via a custom survey at 12 weeks. The proportion of participants experiencing adverse events, and the nature of the adverse events, will be described.

#### Pain at other sites (hips, knees, ankles, other foot sites, spine)

This is scored as the number of other pain sites, and severity of pain at each site using NRSs described above (terminal descriptors ‘no pain’ (score = 0) and ‘worst pain possible’ (score = 10)).

#### Treatment adherence

Participants record the hours/day they wore their orthoses/insoles and footwear daily for the final 7 consecutive days of weeks 4, 8 and 12 in log books. Adherence will be calculated as the average percentage of time spent wearing the study orthoses/insoles when wearing shoes. Participants will be classified as adherent if they wear their insoles for an average of at least 70% of the time spent wearing shoes. Participants in both groups also rate their adherence to wearing their allocated insoles using an 11-point NRS (from ‘not at all’ (score = 0) to ‘completely as instructed’ (score = 10)) at the 12-week follow-up. Finally, participants also indicate if they ceased wearing their study insoles over the course of the study on a categorical scale (yes or no) at the 12-week follow-up. Those who respond ‘yes’ are required to describe why and when they ceased wearing their allocated insoles, and this information will be reported descriptively.

#### Descriptive measures

Descriptive measures are assessed at baseline unless otherwise indicated. These include age, height, weight, body mass index, gender, duration of symptoms, current employment status and expectation of treatment outcome (assessed on a 5-point ordinal scale from “no effect at all” to “complete recovery”). Credibility of the allocated insole is assessed via the Credibility/Expectancy Questionnaire (CEQ) [37] during the clinic visit immediately after receiving the allocated insole. First MTP joint radiographic disease severity is measured during radiographic screening using a validated atlas [23].

#### Clinical data

At the initial clinical visit, the podiatrist assesses foot posture with the participant standing barefoot using the foot posture index (FPI) [29]. The FPI is scored between − 12 (severely supinated) to + 12 (severely pronated). The number and proportion of participants with a supinated (− 12 to − 1), neutral (0 to + 5) or pronated (scores of + 6 to + 12) foot posture will be reported. Hallux valgus severity is assessed using the Manchester scale [24]. Big toe joint dorsiflexion range of motion is measured with the participant in supine using a goniometer [38]. Orthoses modifications (e.g. use of a first ray cut out/extension and/or varus wedge) and footwear type and/or advice are documented using a custom table at the initial clinical visit.

### Sample size calculations

The minimal clinically important difference (MCID) in our primary outcome is a change in NRS walking pain of 1.8 out of 10 units [39]. We assume a between-participant standard deviation of 2.5 and a baseline to 12-week correlation of 0.3 using our pilot RCT data. Using ANCOVA adjusted for baseline score, we need 37 participants per arm to achieve 90% power to detect the MCID in pain. Therefore, we will recruit 44 people per arm in total (*n* = 88) to allow for 15% attrition. We have not adjusted the sample size for clustering because podiatrists treat a similar proportion of participants from each group.

### Statistical analyses

A study biostatistician blinded to group allocation will analyse the data. Main comparative analyses between groups will be conducted using intention-to-treat. We will use multiple imputation if > 5% of primary outcome data is missing. To assess whether insole group influences change in walking pain, differences in mean change in pain (baseline minus follow-up) will be compared between groups using linear regression models adjusted for baseline scores on the NRS walking pain, and the stratifying variable of podiatrist. Analyses for continuous secondary outcomes will be conducted similarly. Between-group improvements in participant-perceived global change will be compared using risk differences, calculated from binomial regression models with a logarithmic link, adjusting for the stratifying variable of podiatrist. If this model fails to converge, logistic regression models will be fit. Complete-case analyses will also be conducted. A sensitivity analysis will be undertaken to investigate the effects of insoles assuming full adherence (defined as 70% of time when shod, based on log book data), using an instrumental variables approach [40].

To explore if the effect of insole group on NRS walking pain is moderated by i) BMI, ii) a composite score for osteophyte or joint space narrowing, or iii) first MTP joint range of motion, interaction terms between insole group and each of these variable will be independently included in regression models for NRS walking pain.

### Timelines

The institutional Human Research Ethics Committee approved the study in June 2019. We started participant recruitment in August 2019 and intended to complete recruitment by May 2020. However, due to the outbreak of the COVID-19 virus, recruitment was suspended in March 2020. Final 12-week follow-up data collection was expected to be completed by August 2020, but this will be revised upon recommencement of recruitment.

## Discussion

This protocol outlines the first RCT comparing the effects of contoured foot orthoses to sham flat insoles on pain and other symptoms in people with first MTP joint OA. Foot orthoses are a promising intervention for people with first MTP joint OA. They have been found to alter the biomechanical function of the first MTP joint and foot, and these biomechanical changes are associated with a reduction in joint pain [14–18]. Furthermore, foot orthoses are already widely used by allied health clinicians such as podiatrists and physiotherapists [20], and adherence to their use is good [9], thus our trial reflects current clinical practice. Foot orthoses are also relatively inexpensive, and our study protocol only requires two clinical visits, so the overall cost of our intervention is modest.

Unlike for hip and knee OA, there are no “joint-specific” clinical guidelines for managing patients with first MTP joint OA, and scant research is available to guide clinical practice. Outcomes from our clinical trial will provide much needed evidence regarding a common biomechanical intervention currently used to manage symptoms in people with first MTP joint OA. This information will assist clinicians treating people with this condition, and may be used in future clinical guidelines for this under-researched patient cohort.

## Data Availability

The datasets used and/or analysed during the current study will be made available from the corresponding author on reasonable request.

## Abbreviations

AQoL: Assessment of Quality of Life
CONSORT: Consolidated Standards of Reporting Trials
FPI: Foot posture index
HREC: Human Research Ethics Committee
NRS: Numerical rating scale
OA: Osteoarthritis
PASE: Physical Activity Scale for the Elderly
RCT: Randomised controlled trial
SPIRIT: Standard Protocol Items: Recommendations for Intervention Trials
